# Analysis of possible drug-drug interactions between ritonavir and other antiretrovirals in a section of the private health care sector in South Africa

**DOI:** 10.4102/phcfm.v1i1.21

**Published:** 2009-06-18

**Authors:** Norah L. Katende-Kyenda, Martie S. Lubbe, Jan H.P. Serfontein, Ilse Truter

**Affiliations:** 1 Department of Pharmacology, Walter Sisulu University, South Africa; 2 School of Pharmacy, North-West University, South Africa; 3 Department of Pharmacy, Nelson Mandela Metropolitan University, South Africa

**Keywords:** antiretroviral drugs, drug-drug interactions, human immunodeficiency virus patients, private health care, ritonavir

## Abstract

**Background:**

The introduction of human immunodeficiency virus (HIV) protease inhibitors (PIs) has led to a dramatic decline in the morbidity and mortality associated with HIV infection. However, the concomitant use of PIs and other antiretrovirals (ARVs) can be complicated by drug-drug interactions (DDIs), adversely affecting levels of PIs.

**Method:**

A quantitative, retrospective drug utilisation study was performed using data obtained from the medicine claims database of a pharmacy benefit management company during 2004, 2005 and 2006. The possible DDIs found among ARVS themselves were identified using the classification by Tatro.

**Results:**

The percentage of ARV prescriptions claimed of the total number of medicine items increased from 1.68% (n = 43 482) during 2004 to 3.18% (n = 51 613) during 2005, then to 4.74% (n = 47 085) during 2006. A total of 1 326, 1 863 and 960 possible DDIs were identified among ARVs themselves for 2004, 2005 and 2006 respectively. Of these, ritonavir (unboosted or boosted) presented with the most possible DDIs, accounting for 74.28% (n = 985) for 2004; 67.90% (n = 1 265) for 2005; and 27.50% (n = 264) for 2006. The highest prevalence of DDIs identified was between ritonavir (unboosted) and saquinavir (n = 974, 5) for 2005 and 2006; followed by indinavir (n = 490, 129, 155) for 2004 to 2006; and efavirenz (n = 274) for only 2004; then ritonavir (boosted), co-formulated as lopinavir/ritonavir, and efavirenz (n = 118, 88, 34) for 2004 to 2006; nevirapine (n = 49, 37) for 2004 and 2005; indinavir (n = 9) for 2004; and saquinavir (n = 22) for 2006.

**Conclusion:**

These findings indicate that concomitant use of PIs such as ritonavir, a potent cytochrome P450(CYP)3A4 enzyme inhibitor, and other ARVs is complicated by possible DDIs and therefore further studies need to be done on the ARV combinations and management of these DDIs.

## INTRODUCTION

In managing human immunodefiency virus (HIV)-1 infection, the current best available route is to achieve both sustained suppression and altered natural history of viral replication in all cellular and body compartments, using highly active antiretroviral therapy (HAART).^1, 2^

The HAART regimens currently recommended as first-line treatment are protease inhibitor (PI) based or non-nucleoside reverse transcriptase inhibitor (NNRTI) based^3^; triple nucleoside reverse transcriptase inhibitor (NRTI)-based regimens are an alternative when PI- or NNRTI-based regimens are unsuitable.^4^ The clinical value of triple-combination antiretroviral (ARV) therapy has been established by a number of large randomised controlled trials showing striking improvements in disease markers, improved survival and diminished disease progression relative to single- and double-agent therapy.^5^

The introduction of HIV-1 PIs has been associated with a dramatic reduction in AIDS-related morbidity and mortality because there are potent ARV agents that, either alone or co-administered with NRTIs, have demonstrated substantial virological and immunological responses sustained over long periods of followup.^6^ Ritonavir is one of the four potent synthetic HIV PIs that have revolutionised HIV therapy.

One of the most challenging issues encountered by providers treating patients with HIV-1 infection is the complex problem of drug-drug interactions (DDIs) associated with HAART. Guidelines for the initial treatment of HIV infection recommend the use of at least three ARVs, each of which is associated with significant DDIs. Either NNRTI-based or PI-based HAART regimens are strongly recommended. Although PIs are preferably employed, there are potent inhibitors of CYP3A4, resulting in possible DDIs that are often very complex. Among the PIs, ritonavir (a so-called booster) is the most employed in combination with other ARVs to enhance plasma drug concentration and, therefore, increase antiretroviral activity.^7^ It is the most potent inhibitor of CYP3A4, therefore is the most likely PI medication to cause DDIs.^6^

A study by Boffito et al.^8^ indicated that the use of boosted double-PI regimens can produce pharmacokinetic interactions; for example, the use of tipranavir/ritonavir with other PIs indicated a significant decrease in plasma concentrations of saquinavir, amprenavir and lopinavir. Therefore, such a combination should be avoided.

Another study performed by the same authors^8^ concluded that when lopinavir/ritonavir was combined with fosamprenavir, the results showed substantially lower fosamprenavir levels than in patients dosed with fosamprenavir/ritonavir alone. As a form of managing the interaction, it was suggested that the dose of fosamprenavir be increased from 700 mg twice daily to 1 400 mg daily without changing the dose of ritonavir, though this combination could lead to a complex bidirectional interaction.^8^ Once again, such a combination should be avoided in clinical practice.

The prevalence of DDIs between ritonavir and other ARVs has not been studied in the private health care sector in South Africa. Therefore, the objective of this study was to determine the prevalence of DDIs between ritonavir and other ARVs prescribed on the same prescription in a section of the private health care sector in South Africa for three consecutive years and suggest possible ways of managing such DDIs in clinical practice.

## METHOD

Permission to conduct the study was granted by the pharmacy benefit management company and the study was approved by the Research and Ethical Committees of the North-West University, Potchefstroom Campus, and Walter Sisulu University, Mthatha Campus. This was a quantitative, retrospective drug utilisation study performed on 43 482, 51 613 and 47 087 ARV prescriptions claimed during 2004, 2005 and 2006 respectively, through the national medicine claims database of a pharmacy benefit management company in a section of the private health care sector of South Africa.

This company is an organisation that manages the benefits of a certain section of medical schemes and insurance companies in South Africa by providing a real-time auditing process to claims from pharmacies and service providers. The medical scheme administrators administered the claiming data of 80, 68 and 36 medical aid schemes during 2004, 2005 and 2006 respectively.^9^ The number of medical schemes covered in 2006 was smaller as compared to 2005 and 2004, as was reflected in the smaller number of ARV prescriptions claimed. There are various pharmacy benefit management companies in South Africa and a medical scheme can decide whether a pharmacy benefit management company should manage its benefits or whether to do so independently.

The database provided information about the trade name of the drug, the National Pharmaceutical Product Interface (NAPPI) code,^1^ the date the prescription was filled, the prescription number, identification numbers for the patient (dependant), physician, pharmacy and medical scheme, the number of the medicine items prescribed and the amount paid by the medical scheme. Dummy membership numbers (randomly allocated by the PBM) were used to prohibit the identification of the patient; thus maintaining anonymity. No specific patient, medical practice, pharmacy or medical scheme could be identified, thus confidentiality of information was maintained throughout the study. Data were analysed by using the Statistical Analysis System^®^ (SAS 9.1).^10^

For the purpose of this study a drug item (medicine item) is defined according to the Medicines and Related Substances Control Act of 1965^1^ as ‘substance intended for use in the diagnosis, cure, mitigation, treatment, modification or prevention of disease, abnormal physical or mental state or the symptoms thereof in man.’ In this research the words drug items are used interchangeably with the words medicine items. In the South African context, a prescription can consist of one or more medicine (or drug) items.

The focus of this study was to determine possible DDIs between unboosted/boosted ritonavir and other ARVs, in a private health care sector in South Africa. The possible DDIs found were classified according to a clinical significance rating, and the formula for the clinical significance ratings of DDIs is described in the form of three degrees of severity, identified as major, moderate and minor.^12^ Drug interactions assigned documentation levels of established, probable or suspected are considered to be well substantiated and have significance ratings of 1 (major), 2 (moderate), 3 (minor) and 4 (major/moderate). These interactions have a probability of occurring, while interactions with significance ratings 5 are not substantiated, having documentation levels of possible or unlikely.

The study population consisted of all ARV prescriptions claimed during 2004 (N = 43 482), 2005 (N = 51 613) and 2006 (N = 47 085). The data consisted of ARV drug names that were classified according to the pharmacological groups as described in the *Monthly Index of Medical Specialties* (MIMS).^13^

The data were obtained directly from the database of the pharmacy benefit management company and analysed without any direct manipulation of the data by the researcher. Certain limitations that could limit the scope of the study were identified. Data were obtained from one medicine claims database, thus limiting external validity, implying that the results can be generalised only to the specific database used as well as to the specific study population. Research was conducted from the viewpoint that all data obtained from the medicine claims database were correct and accurate.

## RESULTS

The data obtained from a medicine claims database during 2004, 2005 and 2006 consisted of 2 595 254, 1 621 739 and 993 804 medicine items of which 43 482, 51 613 and 47 085 were ARV prescriptions claimed during the three years. The percentage of ARV prescriptions claimed increased from 1.68% during 2004 to 3.18% during 2005 and 4.74% during 2006. A total of 1 326, 1 863 and 960 possible DDIs were identified among ARVs themselves for 2004, 2005 and 2006 respectively. Ritonavir (unboosted and boosted) presented with the most possible DDIs, accounting for 74.28% (n = 985) for 2004; 67.90% (n = 1 265) for 2005; and 27.08% (n = 264) for 2006 (see Table 1).

**TABLE 1:** A three-year comparison of the total number of medicine items, ARV prescriptions, DDIs among ARVs and DDIs between ritonavir and other ARVs

Year	Medicine items	ARV prescriptions	DDIs among ARVs	DDIs between ritonavir (unboosted and boosted) and other ARVS
2004	2 595 254	43 482	1 326	985
2005	1 621 739	**51 613**	**1 863**	**1 265**
2006	993 804	47 085	960	264

As observed in Table 1, 2005 presented with the highest number of ARV prescriptions claimed from the database, giving the highest number of DDIs among ARVs themselves and also the highest number of DDIs between ritonavir (boosted and unboosted) and other ARVs. The year 2006 had fewer ARV prescriptions claimed because fewer medical aids were contracted than in 2005, and this explains the decline in DDIs both among ARVs themselves and between ritonavir and other ARVs.

As observed in Table 2, 2005 had the highest number of DDIs between ritonavir (unboosted) and other ARVs, as it was the year with the highest number of ARV prescriptions claimed from the database, followed by 2004 and 2006 respectively. The highest number of DDIs was identified between ritonavir (unboosted) and saquinavir, followed by indinavir, efavirenz and nevirapine. DDIs between ritonavir (unboosted) and saquinavir presented at clinical significance level 3 (minor),^12^ with mild effects and without significantly affecting the therapeutic outcome. DDIs at clinical significance level 2 (moderate)^12^ presented between ritonavir (unboosted) and indinavir, efavirenz and nevirapine – effects may cause deterioration of a patient's clinical status and additional treatment, hospitalisation or extension of stay in the hospital may be necessary.

**TABLE 2:** DDIs between ritonavir (unboosted) and other ARVs for 2004, 2005 and 2006

INTERACTING ARVS	2004		2005		2006	

	(n)	%*	(n)	%*	(n)	%*
**Ritonavir + saquinavir**	-	-	974	85.44	5	2.45
**Ritonavir + indinavir**	490	60.57	129	11.34	155	97.55
**Ritonavir + efavirenz**	274	33.87	-	-	-	-
**Ritonavir + nevirapine**	45	5.56	37	3.25	-	-

**TOTAL**	**809**	**100.00**	**1 140**	**100.00**	**204**	**100.00**

* Percentage was calculated according to the total number of possible DDIs identified in a specific year

The other regimens where most DDIs were identified were between ritonavir (boosted), co-formulated as lopinavir/ ritonavir, and efavirenz (n = 118, 88, 34) for 2004 to 2006; nevirapine (n = 49, 37) for 2004 and 2005; indinavir (n = 9) for only 2004; and saquinavir (n = 22) for only 2006 (see Table 2). All ARVs were interacting at clinical significance level 2 (moderate),^12^ causing deterioration of a patient's clinical status.

As observed in the Table 3, the highest number of DDIs was identified between the boosted ritonavir and efavirenz for the three years, followed by nevirapine, saquinavir and indinavir. All ARVs were interacting at clinical significance level 2 (moderate).^12^

**TABLE 3:** DDIs between ritonavir (boosted) and other ARVs for 2004, 2005 and 2006

INTERACTING ARVs	2004		2005		2006	

	(n)	%*	(n)	%*	(n)	%*
**LPV/RTV + efavirenz**	118	67.05	88	70.40	34	98.21
**LPV/RTV + nevirapine**	49	27.84	37	29.60	-	-
**LPV/RTV + indinavir**	9	5.11	-	-	-	-
**LPV/RTV + saquinavir**	-	-	-	-	22	1.79

**TOTAL**	**176**	**100.00**	**125**	**100.00**	**56**	**100.00**

* Percentage was calculated according to the total number of DDIs identified in a specific year.

## DISCUSSION

The aim of this study was to determine the prevalence of possible DDIs between ritonavir (unboosted and boosted) and other ARVs in a section of the private health care sector, considering that HIV PIs are widely used in combination antiretroviral therapy and that certain characteristics make them prone to clinically significant DDIs with other ARVs. Data for the study were obtained from prescriptions claimed in a section of the private health care sector in South Africa. The study indicated that ARV prescriptions claimed from the database for the three years accounted for 1.92%, 3.38% and 4.73% of the total number of 2 595 254, 1 621 739 and 993 804 prescriptions claimed during 2004, 2005 and 2006 respectively. A total of 1 326, 1 863 and 960 possible DDIs were identified between ARVs themselves for 2004, 2005 and 2006 respectively. Ritonavir (unboosted and boosted) presented with the most possible DDIs, accounting for 74.28% for 2004; 67.90% for 2005; and 27.08% for 2006 (see Table 1).

The relevance of these findings for the three years is that 2004 was the year before the implementation of prescribed minimum benefits (PMBs) in HIV/AIDS in South Africa,^14^ whereas 2005 was the year when PMBs in HIV/AIDS were implemented and by 2006, PMBs were fully functioning. Katende et al.^15^ in their findings stated how the implementation of PMBs in HIV/ AIDS in South Africa had a positive impact on the management of HIV/AIDS with a decrease in the number of DDIs among ARVs, as has been demonstrated in this study for the year 2006. One possible weakness of this study is that in all three years, DDIs were not identified before the patients’ prescriptions were dispensed. Furthermore, there was no direct manipulation of the data by the researchers; therefore, information such as doses of interacting ARVs and dose adjustments in the different combinations was not analysed.

Since the introduction of HAART, the recommended combination therapy in treatment-naïve patients has been based on two different types of combination regimen, namely NNRTI based and PI based, having efavirenz and nevirapine as the preferred NNRTI and ritonavir (unboosted) and ritonavir (boosted) (lopinavir/ritonavir) as the preferred PI. The combination of PIs and NNRTIs is attractive because both groups of drugs have potent antiretroviral efficacy and both are not antagonistic.^16^ The results of the study showed that most DDIs were between ritonavir (PI) and saquinavir (PI), nevirapine (NNRTI), efavirenz (NNRTI) and indinavir (PI) (refer to Table 3). It has been reported that PI-based regimens revolutionised the treatment of HIV infection, leading to strained viral suppression, improved immunologic function and prolonged patient survival.^17^

A randomised study done by Mathais et al.^18^ stated that although a dose of ritonavir 600 mg twice daily is approved for antiretroviral therapy, it is poorly tolerated due to adverse gastrointestinal effects, changes in serum lipids, insulin resistance and lipoatropy. Therefore, for ritonavir to achieve the desired boosting effect, it is used in its lowest dose. It is therefore recommended that ritonavir be used to boost HIV PIs at doses of 100-200 mg once or twice daily; however, it is reported that even at these low doses, there could be adverse clinical effects, laboratory abnormalities and/or patient intolerance.^19^ This demonstrates that even a change to lower doses could lead to adverse effects.

Murphy et al. ^20^ in an open-label, multicentre trial in 190 antiretroviral treatment patients compared the efficacy of lopinavir/ritonavir at doses of 800/200 mg respectively given once daily plus tenofovir and emtricitabine (both NRTIs) versus lopinavir/ritonavir 400/100 mg twice daily plus tenofovir and emtricitabine. Their results revealed that 71% of the patients treated with once-daily lopinavir/ritonavir achieved and maintained virologic suppression (VL < 50 copies/mL) as compared with 65% of the patients treated with a twice-daily dose of lopinavir/ritonavir. This study demonstrated how a once-daily dosing of lopinavir/ritonavir is therapeutically equivalent to twice-daily dosing in antiretroviral-naïve subjects.

The results of the current study show that the highest number of DDIs occurs between ritonavir and saquinavir, presenting with 979 (see Table 2). Saquinavir as the first PI to be marketed in the USA has very unfavourable pharmacokinetics because its efficacy has been very limited as a result of the low and variable plasma concentrations achieved. However, its pharmacokinetics was reported to be improved when combined with ritonavir.^6^ Thus ritonavir proved to enhance the bioavailability and prolong the elimination half-life of saquinavir so that the plasma concentration time/area under the curve (AUC) of saquinavir increased as much as 30- to 50-fold in comparison with saquinavir alone.^21^ Ritonavir in comparison with other PIs produces the largest increase in saquinavir plasma concentrations and thus may increase the adverse effects of saquinavir. The mechanism by which this interaction occurs is possibly decreased first-pass metabolism (CYP3A4) and post-absorbtive clearance of saquinavir. This interaction is of clinical significance and reduced dosages of saquinavir would produce satisfactory plasma concentrations if adverse effects occur. It is not clear how much ritonavir contributes to the antiviral effect of the high concentration of saquinavir because ritonavir is poorly tolerated at high doses and there are only limited pharmacokinetic data on single low doses of ritonavir and saquinavir.

In a Cochrane Review Group for HIV/AIDS, details of six randomised clinical trials^22^ involving saquinavir/low-dose ritonavir (SQV/r) were retrieved. Different doses of the two drugs were administered, although 400 mg of each twice daily was the most common regimen. The results obtained revealed SQV/r 400 mg/400 mg as the most attractive option as it involved the lowest total doses of the drugs and was better tolerated than the alternatives. The WHO Guidelines^22^ recommend SQV/r 1000/100 mg twice daily. Another popular and widely studied regimen is 400 mg saquinavir + 400 mg ritonavir, and other regimens in use and under study include 1 600 mg saquinavir + 100 mg ritonavir once daily.^22^

In the current study the second highest number of DDIs occurs between ritonavir and indinavir (see Table 2). Indinavir is a potent HIV PI; however, it is also extensively and rapidly metabolised by CYP3A.^23^ It is given as an 800-mg dose every eight hours; however, the regimen still results in low and variable minimum concentration values. It is therefore administered with ritonavir to improve the bioavailability and prolong the elimination half-life of indinavir, in this way to reduce the total dose necessary to achieve a potent antiretroviral plasma concentration.^24^ This was reported in a study performed in 39 healthy adult volunteers, on the effect of ritonavir on the pharmacokinetics of indinavir 800 mg every eight hours; for three doses or 400- or 600-mg single doses, ritonavir increased the indinavir plasma concentrations from 21% to 110% every 12 hours.^6^ However, indinavir plasma concentrations may be elevated, increasing the pharmacological and adverse effects of indinavir. This happens through possibly decreased metabolic (CYP3A4) and post-absorbtive clearance of indinavir.^16^ As form of managing this interaction, it is necessary to closely monitor the patients and adjust therapy as needed.

In a randomised trial of 54 patients, Boyd et al.^25^ compared indinavir at 800 mg three times per day versus indinavir and ritonavir at 800 mg and 100 mg respectively twice per day in 50 patients. The results revealed no differences in virological or immunologic outcome after 112 weeks of treatment of PI-naïve patients. These results demonstrated how a change in the dose frequency does not produce any change in either virological or immunological outcome. However, another study^26^ of pretreated subjects receiving indinavir 800 mg and ritonavir 200 mg twice per day demonstrated HIV RNA suppression to < 400 copies/ mL in 17 (58.6%) of 29 subjects at six months and in eight (57%) of 14 subjects at nine months. These results demonstrated how the same dose change results in higher rates of virologic suppression.

The results of the current study show DDIs between ritonavir, a PI, and efavirenz and nevirapine, both NNRTIs. The combination of PIs and NNRTIs has been reported to be attractive because both groups of drugs have potent antiretroviral efficacy and are not antagonistic.^16^ All PIs are substrates of CYP3A4, meaning that their metabolic rate may be altered in the presence of CYP inducers or inhibitors, while NNRTIs are also substrates of CYP3A4 and can act as an inducer (nevirapine), an inhibitor (delavirdine) or a mixed inducer and inhibitor (efavirenz). Thus, these ARVs can interact with each other and others when prescribed together. When ritonavir (unboosted and boosted) is administered with nevirapine, the PI plasma levels and clinical efficacy may be reduced. Nevirapine, the prototype of NNRTI, is primarily an inducer of CYP3A4 enzymes and, therefore, would be expected to interact with the PIs. In a study, the AUC and clinical efficacy for ritonavir was reduced by 10% in the presence of nevirapine when administered to patients with HIV.^27^

In this interaction, an increase in hepatic metabolism (CYP3A4) of ritonavir is suspected. This happens through increased hepatic metabolism (CYP3A4) of the PI.^28^ Therefore, as part of management careful monitoring of ritonavir plasma levels is required. The clinical response of the patient should be observed when nevirapine is started or stopped, and the dosage of ritonavir should be adjusted as needed.

Matthews et al.^29^ in a study of indinavir-ritonavir combinations reported that regimens with ritonavir doses of > 100 mg twice per day were stopped twice as often as regimens with lower doses, primarily because of gastrointestinal intolerance. This study demonstrated how a change in dose may result in a higher rate of adverse events.

In a study^30^ of 20 treatment-experienced recipients of indinavir at 800 mg twice per day plus ritonavir at 100 or 200 mg twice per day, the L90M mutation was identified in nonresponders and partial respondents but not in responders, despite more consistent through levels, as compared with standard indinavir dosing. On the contrary, another study by Campo et al.^31^ suggested that short-term virological suppression (HIV RNA level, < 400 copies/mL) is possible for subjects with previous failure of PI-based treatment with phenotypic resistance to indinavir when treated with indinavir plus ritonavir at 800 mg and 200 mg twice per day. These results demonstrated how the same dose change can result in lower rates of resistance. Because only subtherapeutic levels of ritonavir are achieved with the use of low levels, this fosters the development of viral resistance, and as long as plasma HIV RNA levels are maximally suppressed, the development of resistance to any of the ARVs in the regimen is minimal. It is important to consider the potential for numerous clinical relevant DDIs during treatment with low-dose ritonavir-containing regimens. In this study, possible DDIs were identified between ritonavir and efavirenz. Efavirenz, another NNRTI, also induces its own metabolism. The induction of CYP3A4 by efavirenz results in enhanced metabolism of the PIs. It has been reported that efavirenz slightly but significantly increases ritonavir exposure. The effect on efavirenz pharmacokinetics has been generally found to be insignificant, although according to a report by Fiske et al.^32^ the AUC of efavirenz increases slightly when it is combined with ritonavir. The inhibition of CYP4503A4 by ritonavir partly offsets the enzyme-inducing effects of efavirenz and nevirapine, which may otherwise advise against the combination of PIs and NNRTIs.^33^ This interaction can be managed by giving 100 mg of ritonavir twice daily, thus not fully blocking the enzyme induction.

Lopinavir has a negligible bioavailability and a short half-life when used alone, but it achieves therapeutic concentrations when combined with ritonavir. In addition to the benefits of twice-daily dosing and a reduced dosage burden because of co-formulation of lopinavir and ritonavir, lopinavir plus ritonavir proved to be safe and effective.^34^ The results of this study show that lopinavir/ritonavir were prescribed together with efavirenz in the three-year period, presenting possible DDIs (see Table 3). This is supported by a quantitative, retrospective drug-utilisation study in which possible DDIs were identified between lopinavr/ ritonavir and efavirenz, accounting for 6.77% of DDIs.^35^ Both efavirenz and lopinavir/ritonavir are inhibitors and inducers of CYP-mediated metabolism. Thus a potential DDI with efavirenz may result in an increased or decreased concentration of PIs. Management of this DDI would involve increasing the lopinavir/ritonavir dose by 33% during co-administration with efavirenz, compensating for the enzyme-inductive effect of efavirenz, resulting in reduced lopinavir levels with the standard lopinavir/ritonavir dose of 400/100 mg twice daily.^34^ Despite the availability of newer agents, the wealth of clinical experience with lopinavir/ritonavir^36^ ensures it a prominent place in the ARV treatment armamentarium for years to come.

### Limitations of the study

The following should be taken into consideration when evaluating these results:

- The lack of detailed demographic and clinical (i.e. age, sex and diagnosis or medical history) information on the database – the relevance of the prescribing patterns could therefore not be determined.
- The clinical relevance of the identified DDIs was evaluated according to criteria stated in the literature. No clinical evaluation of the real effects of these interactions could be done. However, the results emphasise the existence of possible DDIs that could lead to severe drug therapy problems. Further research into the usage of ritonavir in combination with other ARVs in the private health care sector should therefore be conducted in South Africa.
- Various combinations of NNRTIs and PIs are acceptable as HAART, with dosage adjustments of PIs, but in this study prescribed daily doses and therefore dosage adjustments were not investigated and therefore the researchers recommend that further research be done.

### Conclusion

In summary, the current standard of care for HIV patients is a triple-therapy regimen, usually consisting of two nucleoside analogues plus a PI. The availability of anti-HIV drugs facilitates many triple therapies. The PIs are extensively metabolised by the CYP 450 enzymes; therefore, drug interactions involving PIs will occur largely as a result of enzyme induction or enzyme inhibition. The results of this study show that ritonavir, a potent inhibitor of CYP3A4, presents DDIs when prescribed with other ARVs, and these can be markedly managed by dose adjustments.
